# Challenges in economic evaluation of public health interventions: the example of surveillance of high risk pancreatic tumour patients

**DOI:** 10.1093/eurpub/ckac129.237

**Published:** 2022-10-25

**Authors:** P Cortesi

**Affiliations:** Research Centre on Public Health, University of Milano-Bicocca, Monza, Italy

## Abstract

Evaluation of public health interventions raises a number of potential challenges in applying and performing economic analyses. The impact of interventions may extend beyond the health sector, and even within the health sector, the value may be difficult to estimate and extended beyond the health improvement of individuals. Screening and surveillance cancer programmes give the possibility to detect patients in a curable and treatable stage. Increasing number of expert recommendations and guidelines of cancer screening and surveillance have been recorded in the last decades. This scenario has increased the uncertainty on the most valuable and affordable approach to adopt. A case study can be provided by the premalignant pancreatic primarily cystic tumours surveillance. American, European and International guidelines for surveillance of asymptomatic cysts are available with significant differences in patients stratification, surveillance intensity (frequency of visits-exams), and duration. Further, real-world data suggest a significant variability in the surveillance approach even within Europe. However, no comparative data on different programmes efficacy are available, neither reliable medium-long term data on cancer risk. The lack of evidence must be considered with methodological issue associated to the inclusion of method to estimate inequalities created by the different programs and the indirect effect of healthcare resources consumption (e.g. CT and MRI) to access and allocation of these resources to other subjects (cancer and no cancer patients). Economic evaluations of surveillance or screening cancer program raise significant challenges, as data availability and methodological approach to apply. This scenario highlight the need of specific recommendation on data quality and type required to assess the programmes value and on methodological approach required (e.g. type of decision analytical models) to provide useful information for public health decision makers.

